# Outcomes of a Quality Improvement Project to Reduce Unnecessary Blood Tests in Beechcroft Regional Child & Adolescent Mental Health Unit, Belfast Trust

**DOI:** 10.1192/bjo.2022.296

**Published:** 2022-06-20

**Authors:** Catherine Gillanders, Francess Doherty, Sarah Fitzsimmons

**Affiliations:** Beechcroft Regional Inpatient Unit, Belfast, United Kingdom

## Abstract

**Aims:**

An estimated 25% of blood tests are unnecessary with an annual cost to the trust of approx. £26.5 million. Aside from the huge financial impact, patients are undergoing unnecessary invasive procedures with detrimental impact on lab flow processes and inappropriate use of Doctor and Nursing Staff time. Some young people have multiple admissions to Beechcroft in a short space of time or bloods checked in A + E prior to transfer are missed and replicated. Longstanding use of blood template terms “Admission bloods” or “Eating Disorder Bloods” has added to the problem. Initial scoping exercise found one young person had 40 blood tests during their admission. AIM STATEMENT: Reduce baseline blood testing of Glucose, Lipids and TFTs by 10% by June 2021

**Methods:**

QI project commenced December 2019 using the IHI Model for Improvement Methodology was promoted by the project team through conversations with staff, unit meetings, email and posters.

Outcome Measure: Total glucose, lipid and TFT blood tests recorded fortnightly for the unit over 18 months

Process Measures: Training as part of new nursing staff induction, reminders in daily nursing handover, number of staff attending Biochemistry liaison meetings

Balance Measures: Reduced blood test costs, reduced unnecessary staff workload

Change Ideas

6 PDSA cycles were implemented

- Separate Bloods Diary for each ward – January 2020
- Blood diary brought into weekly care planning meetings – July 2020
- Education Posters displayed in ward clinical rooms – September 2020
- MDT meeting with Clinical Biochemistry – April 2021
- Junior Doctor to update bloods diary post weekly care-planning – May 2021
- Bloods diary brought to daily nursing handover & dissemination of new monitoring guidelines – June 2021

**Results:**

Glucose tests reduced by 68% with new median of 2.2 instead of 7. Lipids and TFTs median of 10 remains unchanged.

**Conclusion:**

COVID-19 has disrupted monitoring. Fundamental changes made within our service by stopping blood glucose monitoring and using BMs instead has led to significant improvements. We will continue to monitor results following 2 recent change ideas. We hope to include patient feedback moving forward.

